# Nail Reconstruction With Nail Bed Graft From Big Toe: A Case Report

**DOI:** 10.7759/cureus.27884

**Published:** 2022-08-11

**Authors:** Georgia Syrnioti, Mumen Ayyat, Chloe Jean, Huma Ahmad, Nawaiz Ahmad

**Affiliations:** 1 Plastic and Reconstructive Surgery, Brookdale University Hospital Medical Center, New York, USA; 2 Plastic and Reconstructive Surgery, Saba University School of Medicine, New York, USA; 3 Plastic and Reconstructive Surgery, College of Staten Island, New York, USA

**Keywords:** split-thickness graft, sterile matrix, fingertip injury, reconstruction, nail plate

## Abstract

Fingertip injuries with loss of the nail bed can lead to permanent deformities or absent nail formation. This is a case report of a 17-year-old female who sustained a blunt injury to the left second index finger with nail avulsion. The patient underwent nail reconstruction with a split-thickness graft from the sterile matrix of the left great toe. Postoperatively both the donor and the recipient sites appear to be healing appropriately. Since nail reconstruction with toe graft is rarely performed, this case is of particular interest due to its excellent postoperative outcomes.

## Introduction

Fingernail injuries are one of the most common injuries to the hand. Although nail reconstruction is not commonly performed, it is often necessary for both aesthetic and functional purposes, especially when the avulsion of the nail bed and the matrix are involved [1]. Many methods have been described in the literature depending on the type and the extent of the injury. Treatment options include healing by secondary intention, nail bed grafting from the big toe [2-4], microvascular free nail transfer [5-7], extracellular matrix graft after nail bed excision [8], and placement of thenar fascial flap combined with nail grafting [9,10]. We describe a case of nail reconstruction with a nail bed graft from the big toe.

## Case presentation

**Figure 1:**
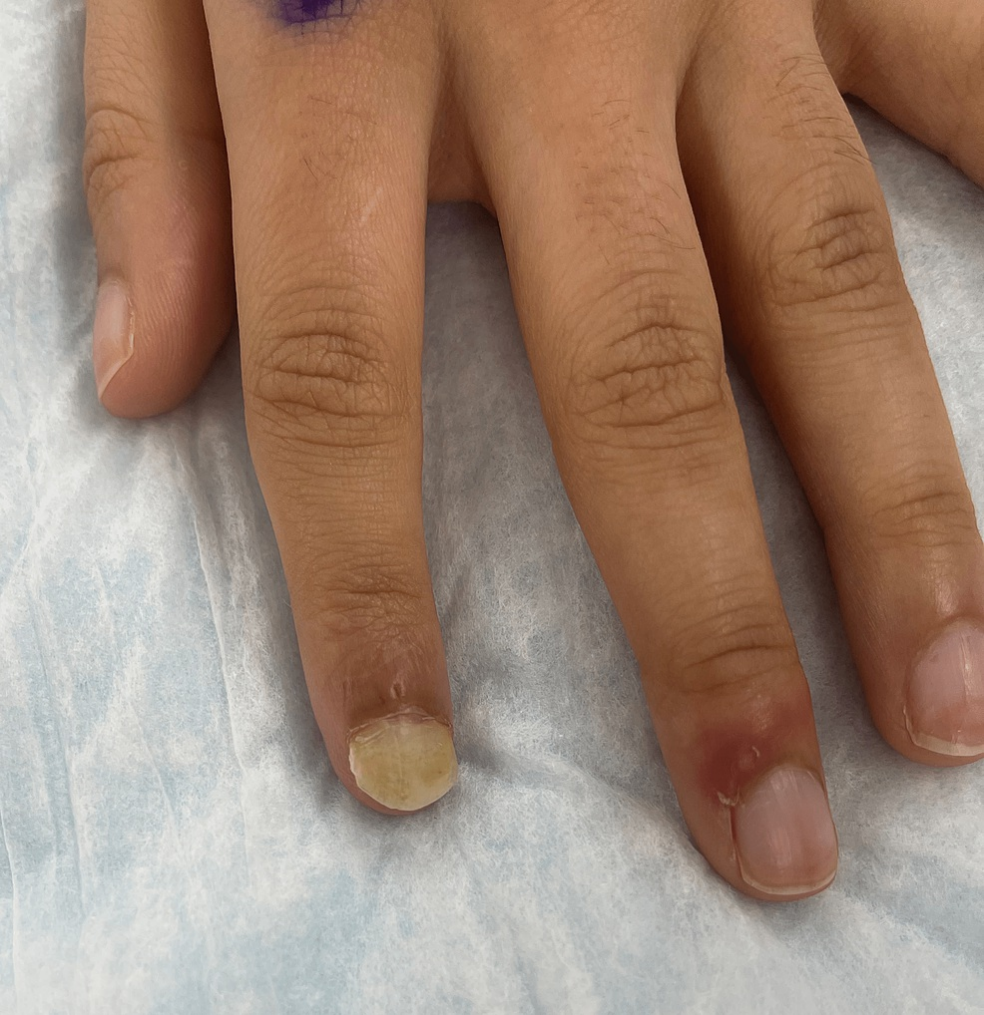
Preoperative appearance of left second finger after nail avulsion

A 17-year-old female with no past medical history presented to the emergency department after sustaining a blunt injury to the left second distal phalanx with nail avulsion. The patient sought reconstruction as the new nail grew deformed and lifted from the nail bed (Figure 1). The patient was taken to the operating room, where removal of the nail plate and debridement of the underlying necrotic tissue were performed. The central portion of the nail plate of the left great toe was lifted, and a portion of the nailbed was harvested using a #11 blade. The harvest nail bed was then secured circumferentially with chromic sutures. Bolster dressing and finger splint was applied to the recipient site. The patient tolerated the procedure well.

**Figure 2:**
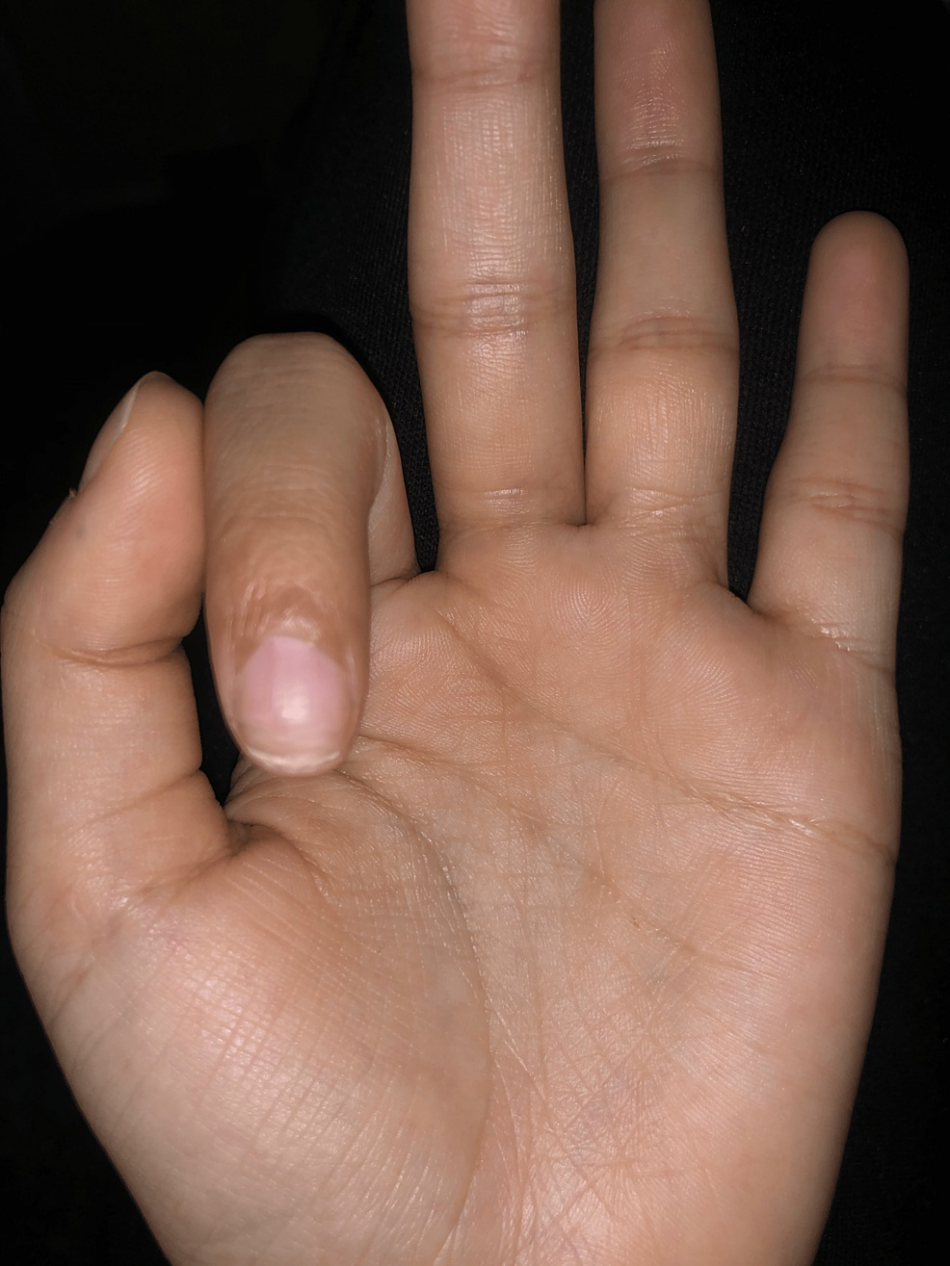
Recipient site four months postoperatively

**Figure 3:**
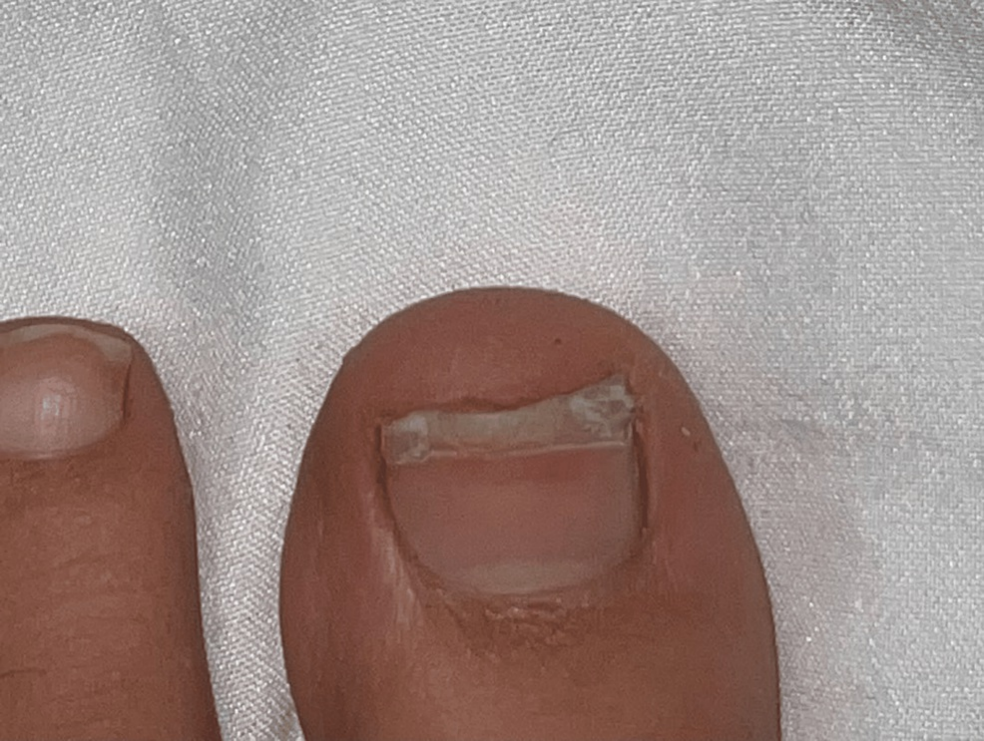
Donor site four months postoperatively

Postoperatively the patient was followed in the outpatient clinic initially every two weeks for the first month and then at one-month intervals for the next three months. Both the donor and the recipient site appear to be healing properly. In fact, four months after the procedure and the new nail growth is graded as excellent (Figures 2, 3).

## Discussion

Fingertip injuries include injuries that are distal to the insertion of the flexor and extensor tendons of the hand. Knowing the anatomy of the fingertip is crucial for the evaluation and management of these injuries [11]. The nail is comprised of the nail plate and the perionychium. The nail plate adheres to the underlying nail bed, which is composed of the proximal germinal matrix and the distal sterile matrix [12,13]. The germinal matrix is responsible for 90% of the nail regeneration, while the sterile matrix anchors the nail plate to the periosteum [14]. Due to the difference in their function reconstruction of the germinal and sterile matrix should be pursued separately. Fingertip injuries with loss of the nail bed can lead to permanent deformities or absent nail formation [15]. Conventional skin grafting of nail bed defects is unlikely to be successful due to the unique characteristics of the germinal and sterile matrix leading to failure of the adherence of the developing nail plate [9,16,17]. Although surgical treatment may not be pursued in small defects, larger defects are managed with excision of the nail plate down to the periosteum and free matrix grafting [18]. In our case, split-thickness matrix grafting was harvested from the great toe leading to good adherence, excellent cosmetic results, and minimal morbidity of the donor site.

## Conclusions

Fingertip injuries continue to be one of the most common injuries to the hand. Challenges regarding their treatment continue to exist. Management is dependent on the extent of the injury, functionality of the affected digit, and the patient’s preferences. In our case, the nail reconstruction offered both excellent cosmetic outcomes and improved the precision pinch ability of the index finger, which plays a major role in the functionality of the hand. 
